# Strain-specific persistence of Burkholderia cenocepacia in the C3HeB/FeJ mouse model of pulmonary infection

**DOI:** 10.1099/jmm.0.002153

**Published:** 2026-04-09

**Authors:** Ziting Chen, Mariana Romero-Gonzalez, Andrea Maser, Ruisi Leng, Lindsay J. Caverly, Marc Hershenson, Ashlee D. Brunaugh

**Affiliations:** 1 Department of Pharmaceutical Sciences, University of Michigan, Ann Arbor, MI, 48109, USA; 2 Department of Pediatrics, University of Michigan Medical School, Ann Arbor, MI, 48109, USA

**Keywords:** antimicrobial resistance, *Burkholderia cenocepacia*, C3HeB/FeJ mice, macrophage activation, persistent pulmonary infection, strain specificity

## Abstract

**Introduction*****.** Burkholderia cenocepacia*, a member of the *Burkholderia cepacia* complex (Bcc), causes severe, treatment-resistant lung infections in individuals with cystic fibrosis and chronic granulomatous disease.

**Hypothesis.** We hypothesized that the C3HeB/FeJ mouse strain, characterized by impaired macrophage-mediated immunity, would be susceptible to persistent infection by clinical Bcc isolates.

**Aim.** To evaluate C3HeB/FeJ susceptibility to various Bcc isolates and characterize the macrophage-intrinsic responses and antibiotic susceptibility within this model.

**Methodology.** C3HeB/FeJ and C57BL/6 mice were compared for susceptibility to pulmonary infection with *B. cenocepacia* AU0728 following intratracheal inoculation. Bacterial persistence and extrapulmonary dissemination were monitored for up to 42 days. Host immune responses were assessed through systemic leucocyte profiling and analysis of bone marrow–derived macrophage (BMDM) function, including intracellular bacterial control, cytokine production and time-resolved cell-death responses. Model breadth was evaluated using three additional clinical Bcc isolates. Finally, the *in vivo* efficacy of high-dose ceftazidime or meropenem was assessed against established pulmonary infection.

**Results.** C3HeB/FeJ mice sustained significantly higher pulmonary burdens of *B. cenocepacia* AU0728 than C57BL/6 mice, resulting in a stable, non-sterilizing infection with extrapulmonary dissemination lasting at least 42 days. *In vitro*, C3HeB/FeJ BMDMs supported higher intracellular bacterial loads and failed to mount effective IFN-γ–dependent intracellular control. Mixed-effects modelling of cell-death responses revealed that both strains initiated early apoptotic signalling following infection; however, this response was attenuated in C3HeB/FeJ macrophages and was not restored by IFN-γ pretreatment. In contrast, late-stage membrane permeabilization increased progressively over time and exhibited strain- and IFN-γ–dependent modulation at later stages of infection. Susceptibility was highly strain-specific: three additional clinical Bcc isolates were rapidly cleared from C3HeB/FeJ lungs. Treatment with ceftazidime or meropenem reduced established pulmonary bacterial burdens by ~1.4–1.5 log₁₀ c.f.u. but did not achieve sterilization.

**Conclusion.** These findings identify C3HeB/FeJ mice as a selectively susceptible host for sustained pulmonary infection by *B. cenocepacia* AU0728 and demonstrate that persistence in this model is strongly strain-dependent. Impaired macrophage-intrinsic IFN-γ–dependent control and stage-specific dysregulation of cell-death responses contribute to bacterial persistence. This immunocompetent mouse model provides a tractable platform for dissecting strain-level virulence mechanisms and for evaluating therapeutic strategies targeting chronic Bcc infection.

**Impact Statement**

*Burkholderia cenocepacia* is a major pathogen in cystic fibrosis, where infection can progress to persistent, treatment-refractory lung disease. Progress in understanding chronic infection has been limited by the lack of immunocompetent animal models that support sustained bacterial persistence without invasive manipulation. Here, we identify the C3HeB/FeJ mouse as a selectively susceptible host for pulmonary infection by *B. cenocepacia*, demonstrating that persistence in this model is highly strain-dependent rather than a general feature of Bcc infection. Susceptibility is associated with impaired macrophage-intrinsic IFN-γ–dependent antimicrobial control and stage-specific dysregulation of macrophage cell-death responses, rather than a global shift in apoptotic or necrotic fate. Despite antibiotic responsiveness, infection was not sterilized by high-dose standard-of-care therapy, reflecting clinical challenges in Bcc treatment. Together, these findings establish a tractable immunocompetent model for dissecting strain-level virulence mechanisms and host immune defects that contribute to chronic *B. cenocepacia* infection and for evaluating therapeutic strategies aimed at reducing persistent bacterial burden.

## Introduction

The *Burkholderia cepacia* complex (Bcc) comprises at least 22 closely related Gram-negative species, several of which are opportunistic pathogens in individuals with immunosuppression or chronic lung disease, particularly cystic fibrosis (CF) and chronic granulomatous disease (CGD) [1,2]. Among these, *Burkholderia cenocepacia* and *Burkholderia multivorans* are the most prevalent, together accounting for ~70% of Bcc infections in people with CF [3,4]. Both species are associated with chronic airway colonization and, in some cases, rapid clinical deterioration, including fulminant necrotizing pneumonia and bacteraemia (cepacia syndrome) [5]. Infection with *B. cenocepacia* is also linked to significantly increased post-transplant mortality in CF, with rates several-fold higher than in patients infected with other Bcc species or uninfected individuals [6]. These clinical challenges underscore the need for scalable animal models that support persistent Bcc infection to enable mechanistic studies of host–pathogen interactions and therapeutic response.

The mechanisms by which Bcc infections establish and persist remain incompletely understood. In CGD, susceptibility is driven by impaired production of reactive oxygen species (ROS) by phagocytes [2]. In CF, mutations in the CFTR gene similarly impair phagocyte function, through defective chloride transport that disrupts the neutrophil respiratory burst [7] and alters macrophage gene expression, resulting in blunted activation [8]. Additionally, CF airways exhibit defective mucociliary clearance due to altered ionic composition and viscoelasticity of the airway surface liquid, creating a permissive extracellular environment for bacterial infection.

Despite distinct aetiologies, CF and CGD both result in impaired intracellular killing by professional phagocytes such as neutrophils and macrophages. This shared vulnerability may allow Bcc to exploit a persistent intracellular niche, contributing to chronic infection. Clinical [9] and laboratory [10] studies have shown that Bcc species can persist intracellularly within both epithelial cells and phagocytes. Among these, macrophages appear to be a particularly important reservoir; experimental models demonstrate intracellular replication of Bcc in macrophages [11], and histopathologic analysis of lungs from transplant and deceased CF patients with Bcc infection reveals predominantly intracellular localization within macrophages [9].

These observations highlight the need for a preclinical model in which macrophage-intrinsic antimicrobial defences are impaired. The C3HeB/FeJ strain, also known as the ‘Kramnik mouse’, fulfils this criterion. These mice exhibit heightened susceptibility and severe inflammatory lung pathology following infection with *Mycobacterium tuberculosis* [12], an intracellular pathogen that primarily replicates within macrophages. This phenotype maps to the sst1 (susceptibility to tuberculosis) locus on chromosome 1, which harbours a non-functional allele of *Ipr1* [13,14]. *Ipr1* encodes a macrophage-specific transcriptional regulator that enhances bacterial clearance through pathways such as autophagy, apoptosis regulation and reactive nitrogen species production. In its absence, macrophages fail to contain intracellular pathogens. Although *Ipr1* is not homologous to the human FAS gene, both have been implicated in regulating macrophage death during intracellular infection, suggesting a potential functional relevance to human disease. Accordingly, C3HeB/FeJ mice are highly susceptible to other intracellular bacteria, including *Listeria monocytogenes* [15], *Chlamydia pneumoniae* [16] and *Mycobacterium avium* [17].

Given the evidence that Bcc can replicate within macrophages, we hypothesized that loss of Ipr1 function would similarly impair intracellular containment of Bcc, enabling chronic infection. While we hypothesized that this defect would be broadly permissive, we found that susceptibility was highly strain-dependent. Of the four isolates tested, only *B. cenocepacia* AU0728 (a representative of the epidemic PHDC/Midwest clone [18,19]) established a stable, predominantly sub-lethal infection in the lungs and extrapulmonary tissues for at least 6 weeks following intratracheal inoculation. Other isolates were rapidly cleared. These findings identify AU0728 as a clinically relevant Bcc strain capable of exploiting macrophage dysfunction to establish persistent infection and demonstrate that C3HeB/FeJ mice offer a tractable model for dissecting strain-dependent host–pathogen interactions and evaluating therapeutic strategies targeting intracellular Bcc persistence.

## Methods

### Mice

WT mice on the C57BL/6J (Stock No. 000664) and C3HeB/FeJ (Stock No. 000658) backgrounds (The Jackson Laboratory, Bar Harbor, ME) were bred and housed under specific pathogen-free conditions in the Unit for Laboratory Animal Medicine at the University of Michigan. All mice used in experiments were at least 6 weeks of age.

### Bacterial strains and growth conditions

*B. cenocepacia* isolates AU0728 and AU33085, and *B. multivorans* isolates AU37410 and AU37358, were originally obtained from the sputum of individuals with CF and were stored in the CF Foundation *Burkholderia cepacia* Laboratory and Repository at the University of Michigan.

For mouse and macrophage infection studies, bacterial strains were cultured on Difco nutrient agar plates (BD Biosciences, Cat. No. 213000) at 37 °C for 72 h. Colonies were then subcultured into BBL cation-adjusted Mueller–Hinton II broth (BD Biosciences, Cat. No. 212322) and incubated overnight at 37 °C with shaking at 125 r.p.m. until reaching mid-logarithmic phase. Cultures were subsequently diluted into fresh broth to an initial OD at 600 nm (OD₆₀₀) of 0.5, measured using an Epoch 2 microplate spectrophotometer (Agilent BioTek). Bacteria were then diluted in sterile PBS (1X PBS without calcium and magnesium; Corning, Cat. No. 21-040 CM) to the desired concentration for downstream applications.

### Mouse infection models

#### General procedures

All intratracheal infections were performed under isoflurane anaesthesia (Fluriso; VetOne/MWI Animal Health, Boise, ID), with mice receiving 50 µl of sterile PBS containing the indicated dose of bacteria by gently inserting a 200 µl pipette tip containing the inoculum into the mouse trachea. Control mice were mock-infected with 50 µl of sterile PBS. At designated time points, mice were euthanized via inhalation of carbon dioxide followed by cervical dislocation, and lungs (and other organs, when applicable) were aseptically harvested. Organs were weighed and homogenized in 1 ml of sterile PBS using a Precellys Evolution tissue homogenizer (Bertin Technologies, Montigny-le-Bretonneux, France). Homogenates were serially diluted in fivefold steps, plated on nutrient agar (BD Biosciences) and incubated at 37 °C for 72 h. Bacterial colonies were counted to determine c.f.u. per organ.

Mice were monitored at 1 and 24 h post-inoculation and daily thereafter for clinical signs of morbidity, including lethargy, ruffled fur, hunched posture and laboured breathing. Animals meeting humane endpoint criteria were euthanized in accordance with the IACUC-approved protocol. Group sizes ranged from 3 to 5 mice per time point; smaller cohorts were used for baseline confirmation of bacterial deposition, and some animals were removed prior to scheduled endpoints in accordance with humane euthanasia criteria. All individual data points are presented in each figure.

#### Acute *B. cenocepacia* AU0728 infection

Adult C57BL/6 and C3HeB/FeJ mice were inoculated with 6×10⁶ c.f.u. of *B. cenocepacia* AU0728. Mice were euthanized at 30 min (baseline) and on days 1, 2 and 3 post-infection (p.i.).

#### Acute infection with additional Bcc isolates

Adult C3HeB/FeJ mice were inoculated with 5×10⁵ c.f.u. of *B. cenocepacia* AU33085 or *B. multivorans* isolates AU37410 and AU37358. Mice were euthanized at 30 min and 3 days p.i. For extended persistence studies with AU37358, additional mice were euthanized on day 7.

#### Chronic AU0728 infection and histopathology

To model chronic infection, adult C3HeB/FeJ mice were inoculated with 5×10⁵ c.f.u. of *B. cenocepacia* AU0728. Two cohorts were used: one with euthanasia at 30 min p.i. and days 1, 2, 3, 7 and 14, and a second with euthanasia at 30 min p.i. and days 7, 15, 28 and 42. At each time point, lungs, spleens and livers were harvested, weighed and processed as described above.

Tissue processing, paraffin embedding, sectioning (5 µm) and haematoxylin and eosin staining were performed by the ULAM Pathology Core at the University of Michigan. Stained sections were examined by a blinded pathologist.

#### Antibiotic treatment in C3HeB/FeJ mice infected with *B. cenocepacia*

Adult C3HeB/FeJ mice were inoculated with 5×10⁵ c.f.u. of *B. cenocepacia* AU0728. At 24 h p.i., mice received two intraperitoneal injections, 6 h apart, of either ceftazidime (144 mg/kg; TCI Chemicals) or meropenem (144 mg/kg; TCI Chemicals). Control mice received sterile PBS. At 48 h p.i., lungs were harvested, homogenized and processed for c.f.u. enumeration as described above.

### *In vitro* macrophage infection assays

#### Monocyte collection and differentiation

Bone marrow–derived monocytes were isolated from adult C57BL/6 and C3HeB/FeJ mice as previously described [20]. Briefly, femurs and tibias were harvested following euthanasia, cleaned of muscle tissue, soaked in 70% ethanol for 3 min and rinsed with sterile PBS. The epiphyses were removed, and bone marrow was flushed from each bone using 2–3 ml of sterile PBS with a 23-gauge needle. Cells were centrifuged at 200 ***g*** for 5 min at 4 °C, and the pellet was resuspended in 3 ml of red blood cell lysis buffer (Thermo Scientific) for 5 min. Cells were then washed and resuspended in Dulbecco’s Modified Eagle Medium supplemented with 10% FBS and 1% penicillin-streptomycin (complete DMEM), followed by another centrifugation step.

The resulting cell pellet was resuspended in complete DMEM containing 20 ng ml^−1^ macrophage colony-stimulating factor (M-CSF; Invitrogen), filtered through a 70 µm cell strainer and seeded at a density of 0.5×10⁶ cells ml^−1^ in 48-well plates for bacterial enumeration or 1×10⁶ cells ml^−1^ in 96-well plates for cell death assays. Cultures were maintained at 37 °C in a humidified incubator with 5% CO₂. On day 4, half of the medium was replaced with fresh complete DMEM containing 20 ng ml^−1^ M-CSF. After three additional days (day 7), cells were washed with PBS and refreshed with complete DMEM.

To confirm successful differentiation, cells were stained with fluorophore-conjugated antibodies against CD11b (BD Biosciences, M1/70) and F4/80 (Biolegends, BM8). Flow cytometry was performed using a Bio-Rad ZE5 Flow Cytometer, and data were analysed with FlowJo version 11. Cells double-positive for CD11b and F4/80 were gated as macrophages.

#### IFN-γ stimulation and macrophage infection

To determine the impact of IFN-γ stimulation on control of intracellular infection, C57BL/6 and C3HeB/FeJ macrophages were incubated for 24 h in either complete DMEM alone or complete DMEM supplemented with 10 ng ml^−1^ murine recombinant IFN-γ (R and D Systems). Media were replaced with antibiotic-free DMEM on the day of infection.

Isolated *B. cenocepacia* AU0728 colonies were inoculated into broth, grown to mid-logarithmic phase and opsonized in 10% naïve mouse serum for 30 min at 37 °C to facilitate complement-mediated uptake by macrophages, before being applied to cells at a multiplicity of infection (MOI) of 1. Infections were carried out for 1.5 h at 37 °C, after which cells were washed four times with sterile PBS and incubated in complete medium containing 125 µg ml^−1^ ceftazidime to eliminate extracellular bacteria. At designated time points (30 min, 16 h and 40 h p.i.), cells were lysed in PBS containing 0.1% Triton X-100 (Thermo Scientific), and supernatants were collected to confirm extracellular clearance. Lysates were serially diluted in PBS, plated on nutrient agar (BD Biosciences) and incubated at 37 °C for 72 h before c.f.u. enumeration.

#### Cytokine quantification (TNF-α ELISA)

Supernatants were collected from macrophages at 0.5, 16 and 40 h p.i. and stored at −80 °C. TNF-α levels were quantified using a murine TNF-α ELISA kit (R and D Systems, DY410-05) following the manufacturer’s instructions. Absorbance was measured at 450 nm, and cytokine concentrations were calculated using a standard curve. The assay’s lower limit of detection was 31.2 pg ml^−1^.

#### Assessment of cell death in BMDMs

To quantify macrophage cell-death responses, bone marrow–derived macrophages (BMDMs) from C57BL/6 and C3HeB/FeJ mice were infected with *B. cenocepacia* AU0728 at an MOI of 1, with or without IFN-γ pretreatment as described above. Following infection, extracellular bacteria were removed, and cells were maintained in medium containing ceftazidime. Mock-infected controls were included for each condition.

Cell death was monitored longitudinally using the RealTime-Glo^™^ Annexin V Apoptosis and Necrosis Assay (Promega) according to the manufacturer’s instructions. Briefly, detection reagent (100 µl per well) was added at the time of infection, and luminescence (reporting early phosphatidylserine externalization) and fluorescence (reporting late membrane permeabilization) were measured at 0, 3, 6, 16, 24 and 40 h p.i. using a Varioskan LUX multimode microplate reader (Thermo Fisher Scientific).

To account for well-to-well variability, luminescence and fluorescence values at each time point were normalized to the corresponding time-zero value for each individual well prior to statistical analysis.

### Quantification and statistical analysis

Statistical analyses were performed using GraphPad Prism (version 10; GraphPad Software) and R (version 4.5.2). Data are presented as mean±sem unless otherwise indicated.

For comparisons of bacterial burden between two groups, unpaired two-tailed Student’s t-tests were used. For comparisons involving more than two groups, one-way ANOVA followed by Tukey’s or Dunnett’s post hoc tests was applied as appropriate. Two-way ANOVA with Sidak’s multiple comparisons test was used for factorial analyses that did not involve repeated measurements.

Time-course cell-death data were analysed using linear mixed-effects models implemented in R (lme4 and lmerTest packages) to account for repeated measurements within individual wells. Models included strain, infection status, IFN-γ treatment and time as fixed effects, with well identity included as a random intercept. Time was treated as a categorical variable to accommodate non-linear kinetics. Statistical significance of fixed effects and interactions was assessed using Satterthwaite’s approximation for degrees of freedom. Post hoc contrasts and estimated marginal means at selected time points were computed using the emmeans package with Sidak correction for multiple comparisons.

## Results

### *B. cenocepacia* AU0728 establishes a sustained, chronic infection in C3HeB/FeJ mice

Initial characterization focused on the acute dynamics of infection. Adult C57BL/6 and C3HeB/FeJ mice were intratracheally inoculated with 6×10^6^ c.f.u. of the clinical isolate *B. cenocepacia* AU0728 (a representative of the Midwest epidemic clone [18,19]). Lung bacterial burden was quantified at 30 min post-inoculation (baseline) and 24, 48 and 72 h p.i.

**Fig. 1.**
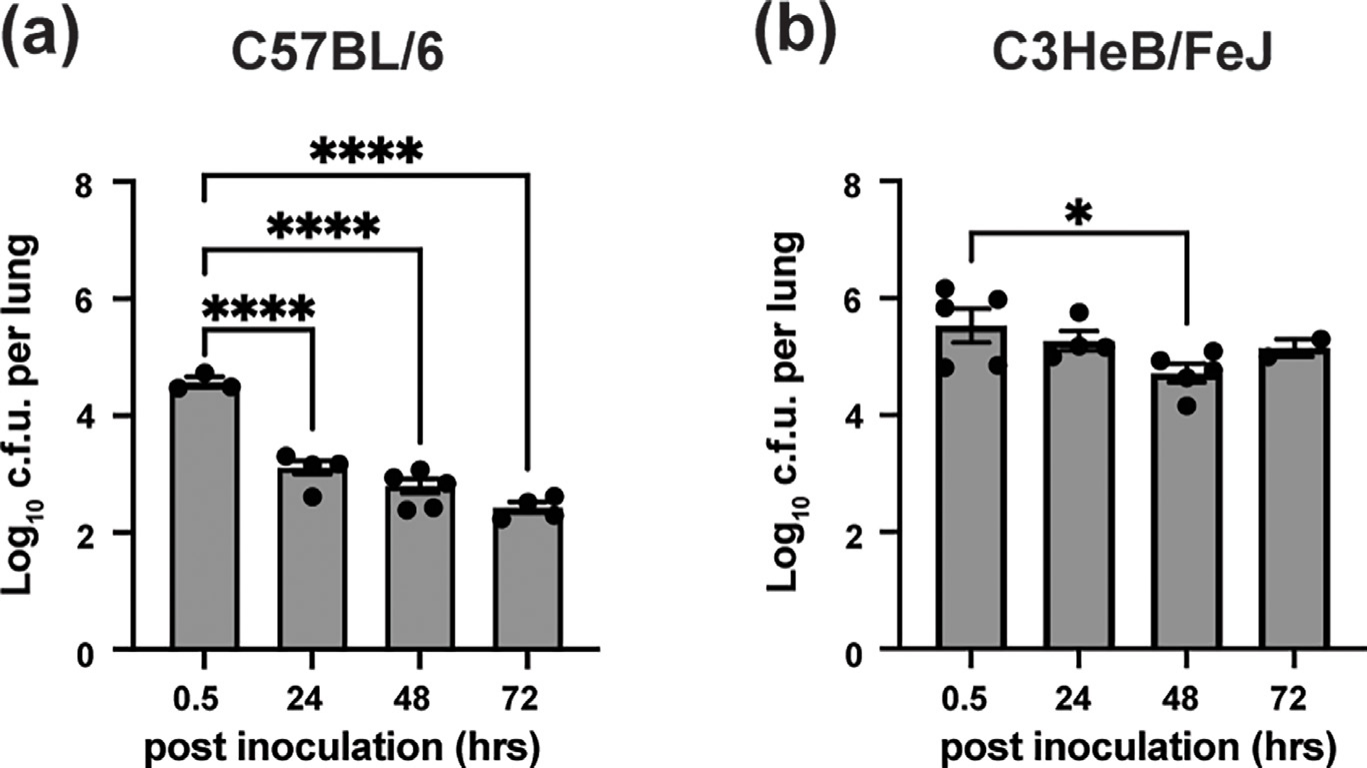
C3HeB/FeJ mice fail to clear *B. cenocepacia* AU0728 during early infection. C57BL/6 and C3HeB/FeJ mice were intratracheally inoculated with 6×10⁶ c.f.u. of *B. cenocepacia* AU0728. Lung bacterial burden was quantified at 0.5, 24, 48 and 72 h post-inoculation (p.i.). (**a**) In C57BL/6 mice, bacterial burden declined significantly over time. (**b**) In contrast, C3HeB/FeJ mice maintained a stable lung bacterial load throughout the 3-day experiment. Bars represent mean±sem; individual mice are shown as dots. *P* values were determined by one-way ANOVA with Dunnett’s post-test versus baseline/0 p.i.

At the 30 min baseline, C3HeB/FeJ mice retained significantly higher bacterial loads (5.52±0.24 log_10_ c.f.u.) compared to C57BL/6 mice (4.56±0.14 log_10_c.f.u.), suggesting an immediate defect in early mechanical or innate clearance mechanisms. In C57BL/6 mice, bacterial counts declined sharply to 3.06±0.31 log_10_ c.f.u. by 24 h (*P*<0.0001), with further reductions observed 48 and 72 h post-inoculation (Fig. 1a). In contrast, C3HeB/FeJ mice showed no significant change between 30 min post-inoculation and 24 h post-inoculation (5.27±0.33 log_10_ c.f.u.; *P*=0.7683), and counts remained stable through 72 h when the study was concluded (Fig. 1b). Notably, 3 of 16 infected C3HeB/FeJ mice were discovered dead at the 72 h time point. Due to this acute lethality, subsequent experiments utilized a reduced inoculum of 5×10^5^ c.f.u. to establish a predominantly sub-lethal infection.

**Fig. 2.**
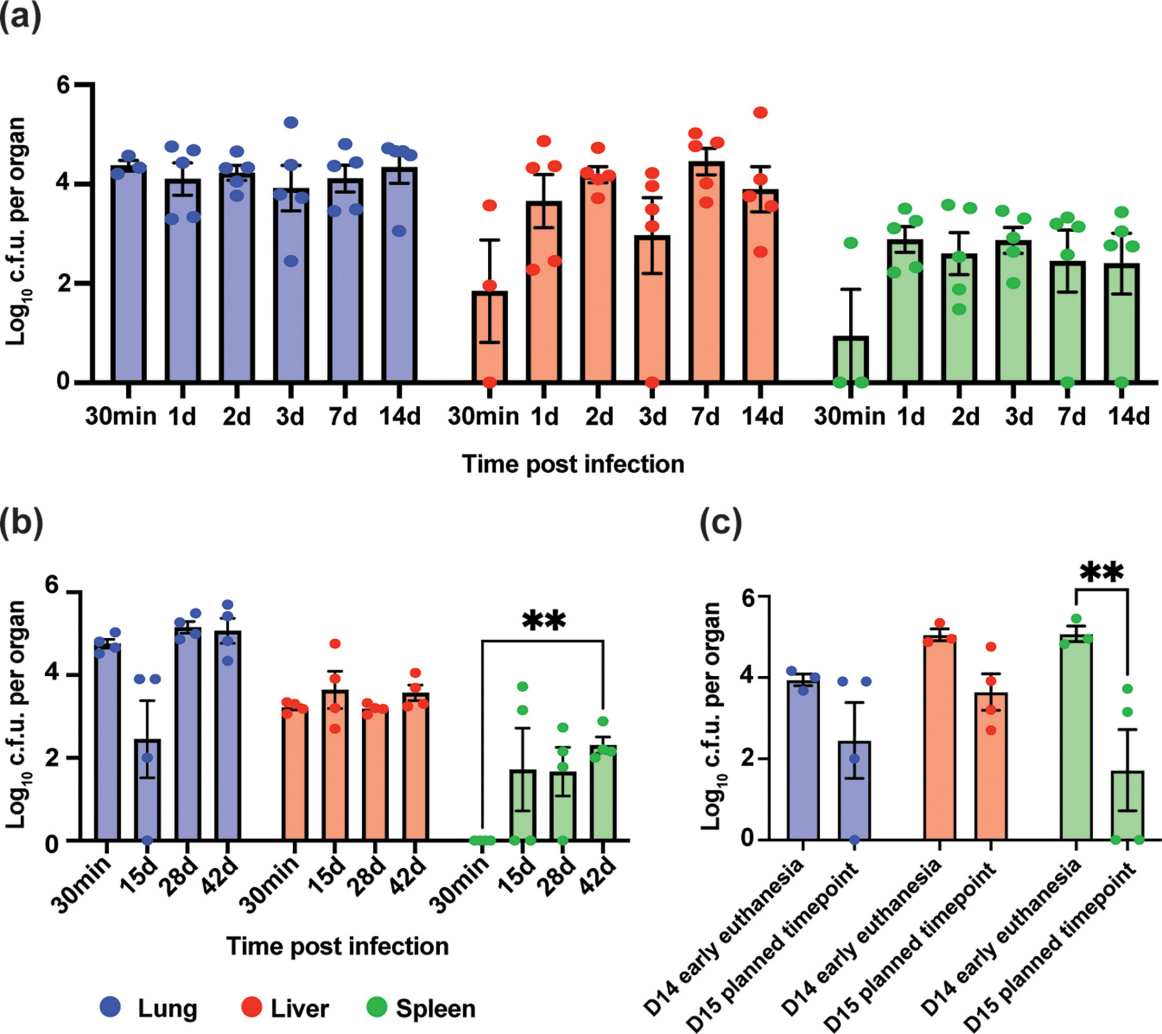
*B. cenocepacia* AU0728 establishes chronic pulmonary and extrapulmonary infection in C3HeB/FeJ mouse strain for at least 6 weeks. (**a**) Lung, liver and spleen c.f.u. were quantified at multiple timepoints over a 14-day infection period. Bacterial loads increased rapidly and persisted in all organs. No significant differences were detected within organ groups over time (mixed effects model with Tukey’s multiple comparisons), though organ effects were significant (*P*<0.0001). (**b**) In an independent study, mice were followed to 42 days p.i. A significant increase in spleen c.f.u. was observed at day 42 compared to baseline (two-way ANOVA with Sidak’s multiple comparisons; *P*=0.0028). (**c**) At day 14 p.i., three mice were euthanized early due to clinical signs. These animals had elevated spleen c.f.u. compared to animals euthanized at the planned endpoint (two-way ANOVA with Sidak’s multiple comparisons; *P*=0.0122). Each dot represents one mouse; bars indicate mean±sem.

To evaluate the long-term stability of the model, C3HeB/FeJ mice were inoculated with a reduced dose of 10^5^ c.f.u. of *B. cenocepacia* AU0728. In an initial 14-day study (Fig. 2a), lung bacterial burdens established at ~4.5 log_10_ c.f.u. at 30 min post-inoculation (baseline) and remained stable through day 14, indicating persistent infection without significant clearance. As expected for intratracheal inoculation models, some variability was observed at this early baseline time point, reflecting differences in initial bacterial deposition within the lung. In the spleen and liver, bacterial burdens rose between baseline and day 1, suggesting early extrapulmonary dissemination, and remained detectable in all organs throughout the 2-week period (Fig. 2a).

To determine the limits of persistence, a second independent cohort was monitored for 42 days (Fig. 2b). Lung burdens remained steady for the full 6-week course. A significant increase in spleen bacterial burden was observed between baseline and day 42 (*P*=0.0028; two-way ANOVA with Sidak’s multiple comparisons), confirming that the infection is not only chronic but capable of progressive systemic dissemination.

While the infection was predominantly sub-lethal at this inoculum, 3 of 18 mice exhibited clinical signs of illness (e.g. lethargy and ruffled fur) at day 14 and were euthanized in accordance with humane endpoints. Post-mortem analysis revealed significantly elevated bacterial burdens in the spleens of these animals compared to those surviving to the planned day 15 endpoint (*P*=0.0122; Fig. 2c), suggesting that a subset of C3HeB/FeJ mice may fail to control systemic spread, leading to a lethal outcome.

**Fig. 3.**
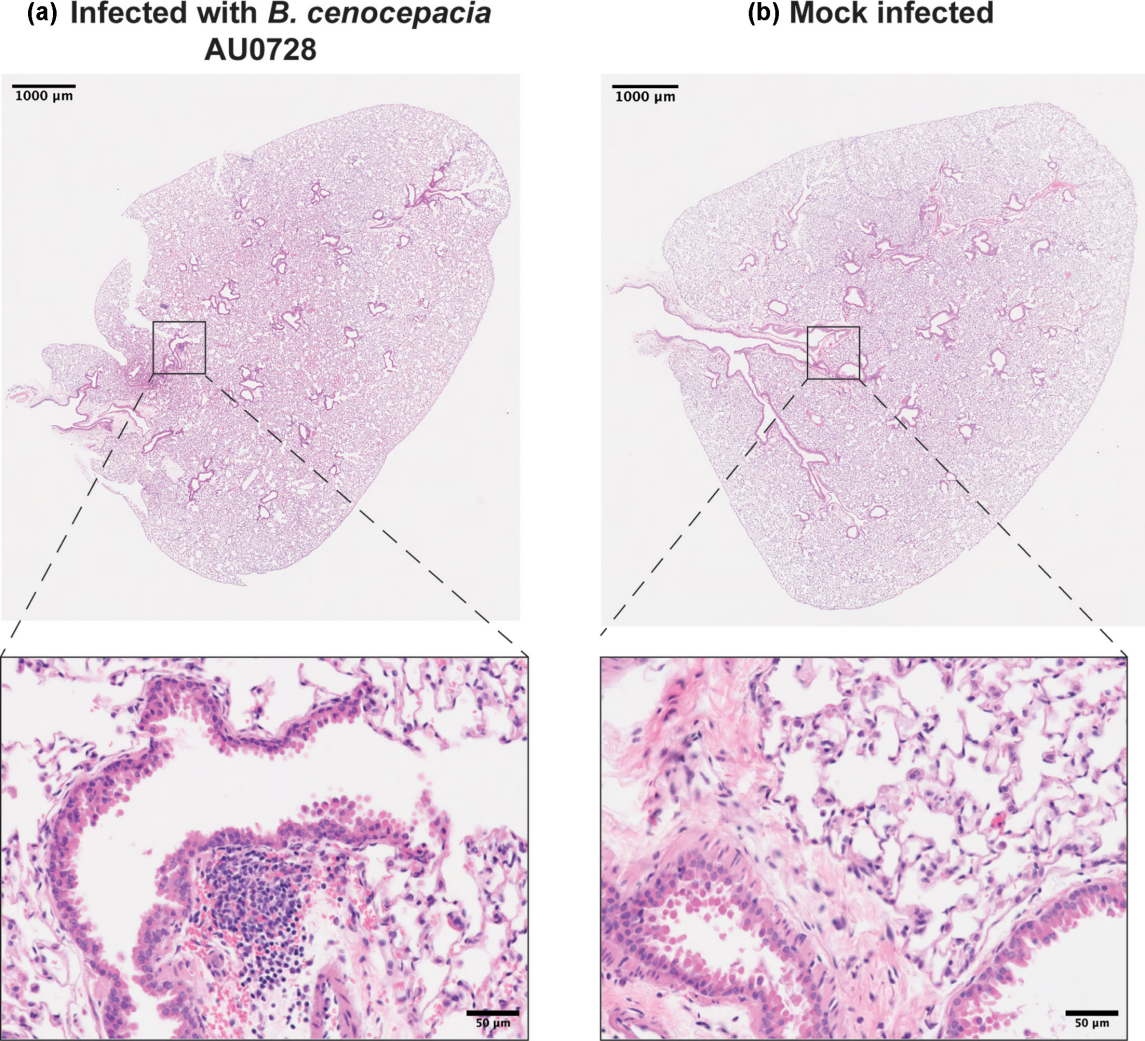
Representative lung histology at 42 days p.i. (**a**) Lung from *B. cenocepacia* AU0728-infected mouse showing multifocal peribronchiolar inflammatory infiltrates. Higher-magnification view reveals infiltrating macrophages, lymphocytes and neutrophils. (**b**) Lung from mock-infected mouse displaying intact airway epithelium and preserved alveolar architecture without inflammatory infiltration. Scale bars: 1,000 µm (overview) and 50 µm (inset).

Histological analysis of lung tissue at day 42 revealed multifocal infiltrates of macrophages, lymphocytes and neutrophils adjacent to bronchioles and vessels, consistent with chronic inflammation (Fig. 3). Unlike the extensive necrosis reported in C3HeB/FeJ tuberculosis models, lesions here were cellular rather than necrotic. No overt histopathological differences were observed in spleen and liver tissue in infected versus mock-infected mice (Fig. S1, available in the online Supplementary Material). No significant changes in organ weights were observed relative to baseline (Fig. S2).

### Susceptibility is associated with impaired macrophage-intrinsic IFN-γ–dependent immunity

To investigate host factors associated with susceptibility to *B. cenocepacia* persistence, we next focused on macrophage-intrinsic immune responses. Given prior evidence that *B. cenocepacia* can replicate within macrophages and that C3HeB/FeJ mice harbour a defect in macrophage-mediated antimicrobial programmes, we examined whether this defect was reflected at the cellular level.

**Fig. 4.**
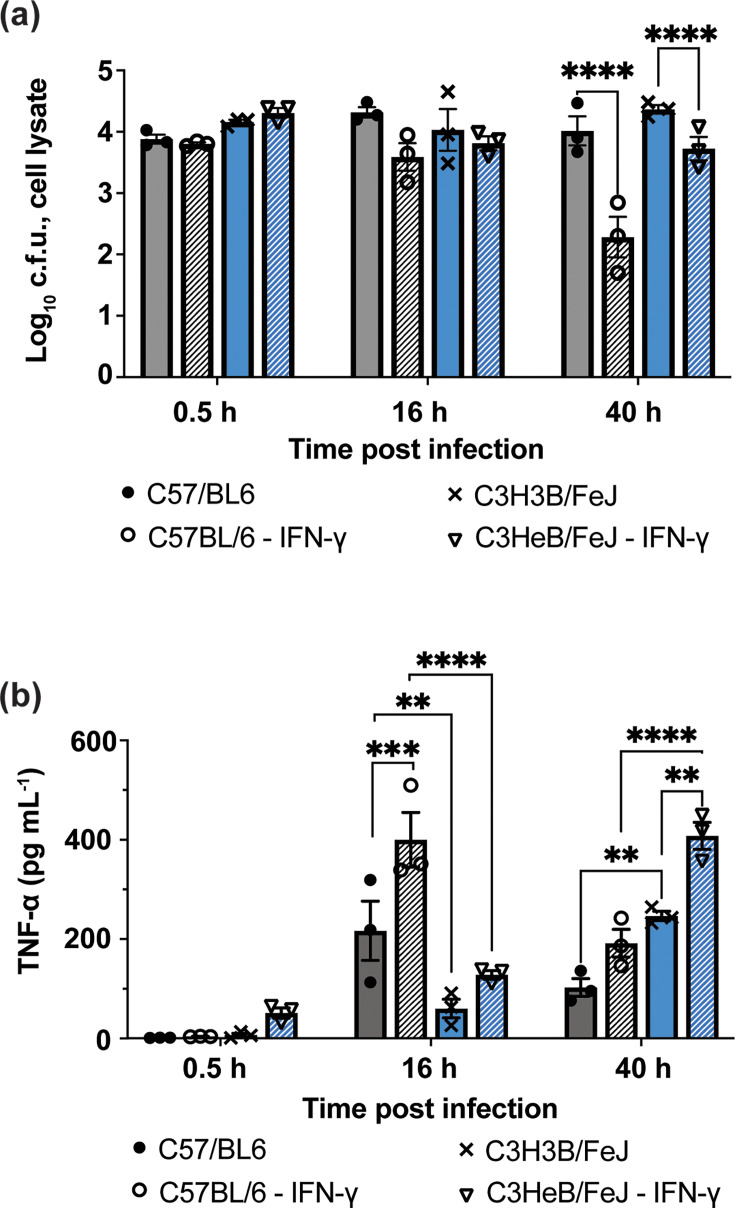
IFN-γ–dependent intracellular control of *B. cenocepacia* AU0728 is impaired in C3HeB/FeJ macrophages. (**a**) Intracellular bacterial burden in bone marrow–derived macrophages (BMDMs) from C57BL/6 and C3HeB/FeJ mice at 0.5, 16 and 40 h p.i. with or without IFN-γ pretreatment. (**b**) TNF-α concentrations in corresponding macrophage culture supernatants measured by ELISA. Bars represent mean±sem; points represent individual biological replicates. Statistical significance was assessed by two-way ANOVA followed by Sidak’s multiple comparisons test. *P<0.05; **P<0.01; ***P<0.001; ****P<0.0001. Macrophage cell-death responses were monitored in parallel and are presented in Fig. 5.

Bone marrow–derived macrophages (BMDMs) were generated from uninfected C57BL/6 and C3HeB/FeJ mice, and successful differentiation was confirmed by CD11b/F4/80 co-expression (Fig. S3). During early infection, intracellular replication of *B. cenocepacia* AU0728 did not differ significantly between macrophages from the two strains. In contrast, IFN-γ priming resulted in a time-dependent restriction of intracellular bacterial burden in C57BL/6 macrophages, with a reduction becoming statistically significant by 40 h p.i., following an earlier trend toward decreased intracellular burden. This IFN-γ–dependent control was absent in C3HeB/FeJ macrophages, which failed to reduce intracellular bacterial burden at any time point examined (Fig. 4a), indicating a strain-specific impairment in IFN-γ–mediated intracellular control.

**Fig. 5.**
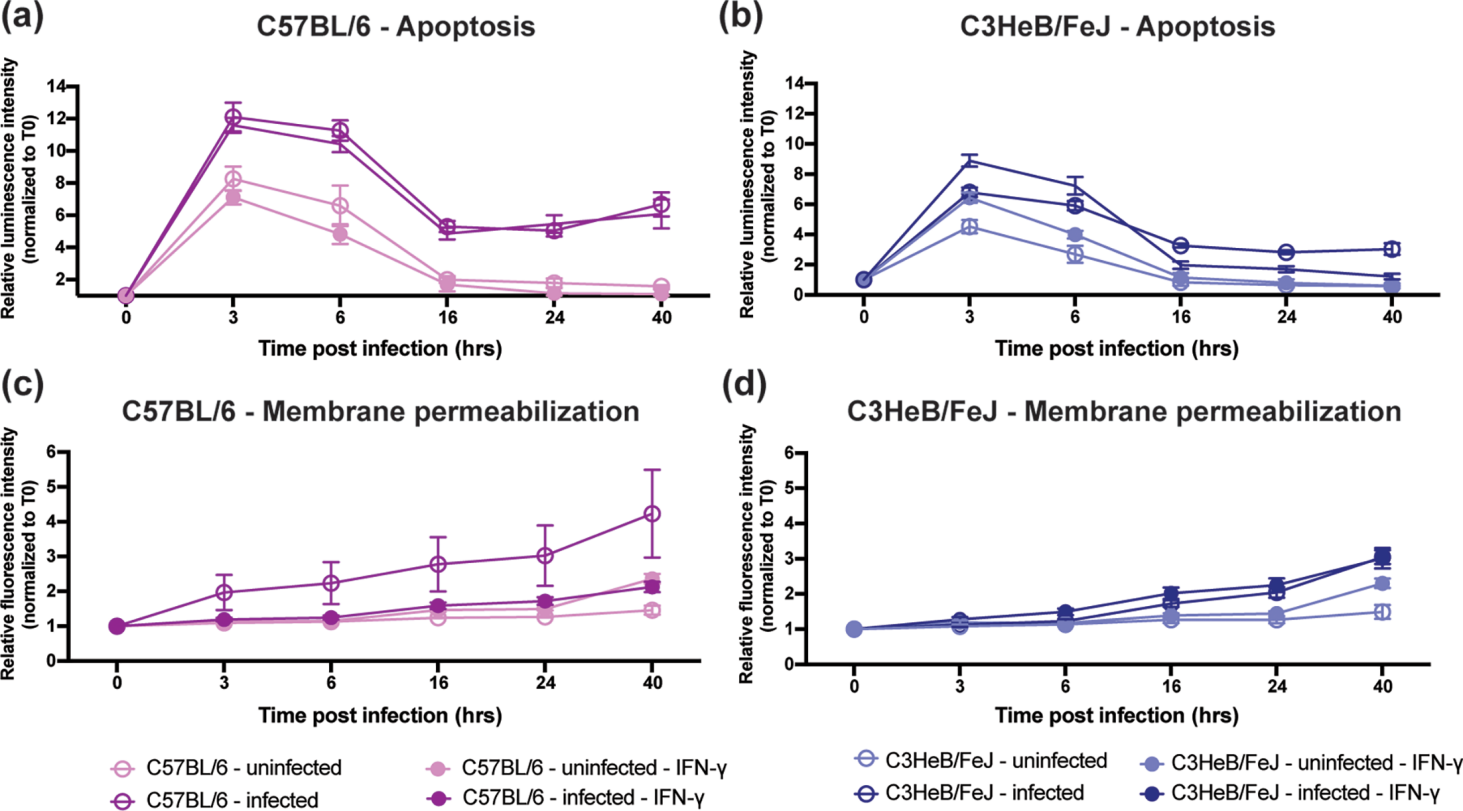
Stage-specific modulation of macrophage cell-death responses during *B. cenocepacia* AU0728 infection. Bone marrow–derived macrophages (BMDMs) from C57BL/6 and C3HeB/FeJ mice were infected with *B. cenocepacia* AU0728 (MOI 1) in the presence or absence of IFN-γ pretreatment, and cell-death responses were monitored over time. (a, b) Early apoptotic signalling was assessed by luminescence and normalized to each well’s baseline (**T0**). Both C57BL/6 and C3HeB/FeJ macrophages exhibited a rapid, transient increase in luminescence following infection, peaking at 3–6 h p.i. and declining toward baseline thereafter, with a reduced magnitude in C3HeB/FeJ macrophages. IFN-γ pretreatment did not substantially alter the early apoptotic trajectory in either strain. (c, d) Late-stage membrane permeabilization was assessed by fluorescence and normalized to T0. In contrast to luminescence, fluorescence increased progressively over time in infected macrophages, consistent with delayed loss of membrane integrity. IFN-γ pretreatment modulated late fluorescence responses at later time points in a strain-dependent manner. Data are shown as mean±sem.

To further assess macrophage activation dynamics, we examined TNF-α secretion over the course of infection. TNF-α induction was strongly time-dependent and differed significantly by experimental group, consistent with distinct activation kinetics. C57BL/6 macrophages mounted a robust TNF-α response by 16 h p.i., whereas TNF-α induction in C3HeB/FeJ macrophages was attenuated at this early time point and increased only at later stages of infection. IFN-γ treatment modulated TNF-α output in both strains but did not restore early TNF-α induction in C3HeB/FeJ macrophages, indicating delayed or dysregulated inflammatory activation rather than an effective early response (Fig. 4b).

To determine whether the impaired IFN-γ–dependent intracellular control observed in C3HeB/FeJ macrophages was associated with altered cell-death dynamics, we quantified apoptotic and necrotic signalling over the course of infection using complementary luminescence- and fluorescence-based assays (Fig. 5). Signals were normalized to each well’s baseline (T0) to isolate infection- and treatment-induced dynamics independent of initial cell number.

Mixed-effects modelling of luminescence revealed that both C57BL/6 and C3HeB/FeJ macrophages initiate an early apoptotic response following infection, characterized by a pronounced and transient increase in luminescence at 3–6 h p.i. Across experimental conditions, infected macrophages exhibited a robust early luminescence peak (3 and 6 h: *P*<0.0001), followed by a decline toward baseline at later time points, consistent with transient activation of early programmed cell-death pathways. Infection significantly amplified this early apoptotic signal over time (infection×time interaction; 3 and 6 h: *P*<0.0001), indicating that apoptotic initiation is a conserved early response to bacterial uptake.

Despite this shared early response, the magnitude of apoptotic signalling differed significantly by host strain. Compared with C57BL/6 macrophages (Fig. 5a), C3HeB/FeJ macrophages displayed a markedly attenuated luminescence response at both 3 and 6 h p.i. (strain×time interaction; 3 h: *P*=0.0012; 6 h: *P*=0.0007), indicating impaired early apoptotic activation in the susceptible strain (Fig. 5b). While weak strain×IFN× time interactions were detectable at early time points, IFN-γ did not meaningfully alter the early apoptotic trajectory in either strain.

We next examined fluorescence, which reports late-stage membrane permeabilization associated with necrotic cell death. In contrast to the transient luminescence response, fluorescence increased progressively over time in infected macrophages. Mixed-effects modelling revealed a strong infection-by-time interaction beginning at 6 h p.i. (6 h: *P*=0.036; 16 h: *P*=0.0036; 24 h: *P*<0.001; 40 h: *P*<0.0001), indicating cumulative membrane damage during ongoing infection.

IFN-γ significantly modulated this late-stage fluorescence response at later time points, as reflected by a significant infection×IFN×time interaction at 24 and 40 h p.i. (24 h: *P*=0.039; 40 h: *P*<0.0001). In contrast to early apoptotic signalling, IFN-γ effects on fluorescence were temporally restricted to later stages of infection.

Strain-dependent differences in fluorescence emerged only at late time points and only in the context of IFN-γ treatment. At 40 h p.i., a significant strain×infection×IFN×time interaction was observed (*P*=0.040), indicating differential IFN-γ–modulated execution of late-stage cell death in C57BL/6 versus C3HeB/FeJ macrophages (Fig. 5c, d).

### Most Bcc isolates are rapidly cleared from C3HeB/FeJ lungs

**Fig. 6.**
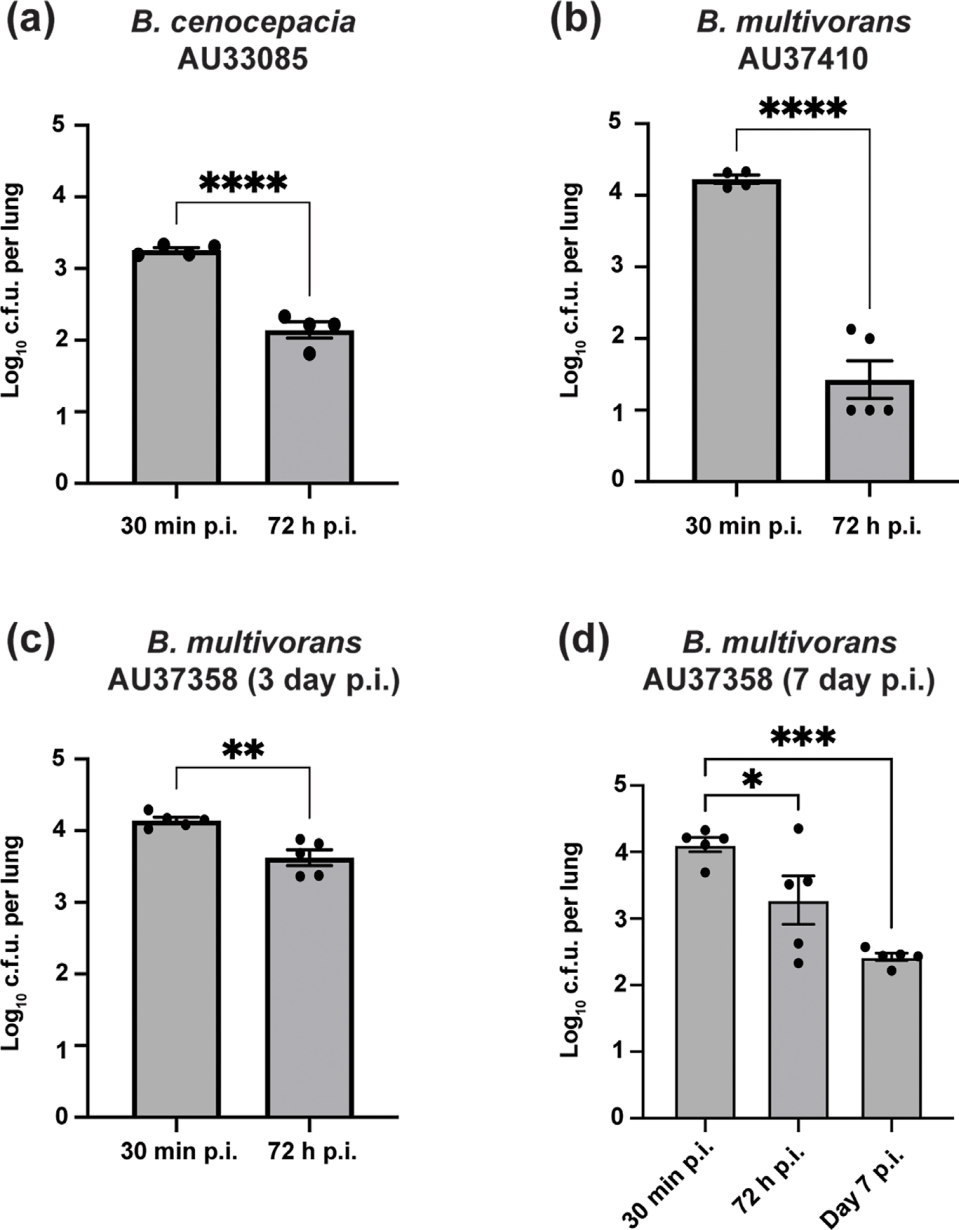
Most Bcc isolates are cleared from the lungs of C3HeB/FeJ mice. (**a**) *B. cenocepacia* AU33085 and (**b**) *B. multivorans* AU37410 were rapidly cleared by day 3 p.i. (c, d) *B. multivorans* AU37358 showed partial reduction by day 3 and further decline by day 7. Baseline indicates 30 min post-inoculation. Bars show mean±sem; dots represent individual mice. *P* values were calculated by unpaired t-test versus baseline (**a–c**) and one-way ANOVA with Dunnett’s multiple comparisons test (**d**). *p < 0.05; **p < 0.01; ***p < 0.001

To assess whether persistence in C3HeB/FeJ mice is a general property of Bcc strains, we infected mice with 5×10⁵ c.f.u. of three additional clinical isolates: *B. cenocepacia* AU33085 and *B. multivorans* isolates AU37410 and AU37358. Both *B. cenocepacia* AU33085 and *B. multivorans* AU37410 were rapidly cleared, with lung bacterial burdens declining from 3.26±0.07 to 2.15±0.22 log₁₀ c.f.u. and from 4.23±0.11 to 1.43±0.59 log₁₀ c.f.u., respectively, by day 3 p.i. (Fig. 6a, b; *P*<0.0001).

*B. multivorans* AU37358 showed a modest reduction by day 3 (4.15±0.10 to 3.63±0.10 log₁₀ c.f.u.; *P*=0.0022), followed by a more pronounced decline by day 7 p.i. (2.42±0.10 log₁₀ c.f.u.; *P*=0.0003; Fig. 6c, d). Thus, among the isolates tested, only *B. cenocepacia* AU0728 displayed sustained early persistence in the lungs of C3HeB/FeJ mice. These findings indicate that susceptibility in this host background is not broadly permissive to Bcc infection but instead reflects a strain-specific capacity to evade or exploit impaired macrophage-intrinsic defences.

### Pulmonary *B. cenocepacia* AU0728 infection in C3HeB/FeJ mice is reduced by standard-of-care antibiotics

To evaluate *in vivo* antibiotic responsiveness, we first confirmed that AU0728 is susceptible to ceftazidime and meropenem by microbroth dilution. C3HeB/FeJ mice were infected intratracheally with 1×10⁵ c.f.u. and treated 24 h later with two intraperitoneal doses of either ceftazidime or meropenem (144 mg kg^−1^ per dose, administered 6 h apart). This dosing strategy was selected based on allometric scaling and pilot susceptibility data.

**Fig. 7.**
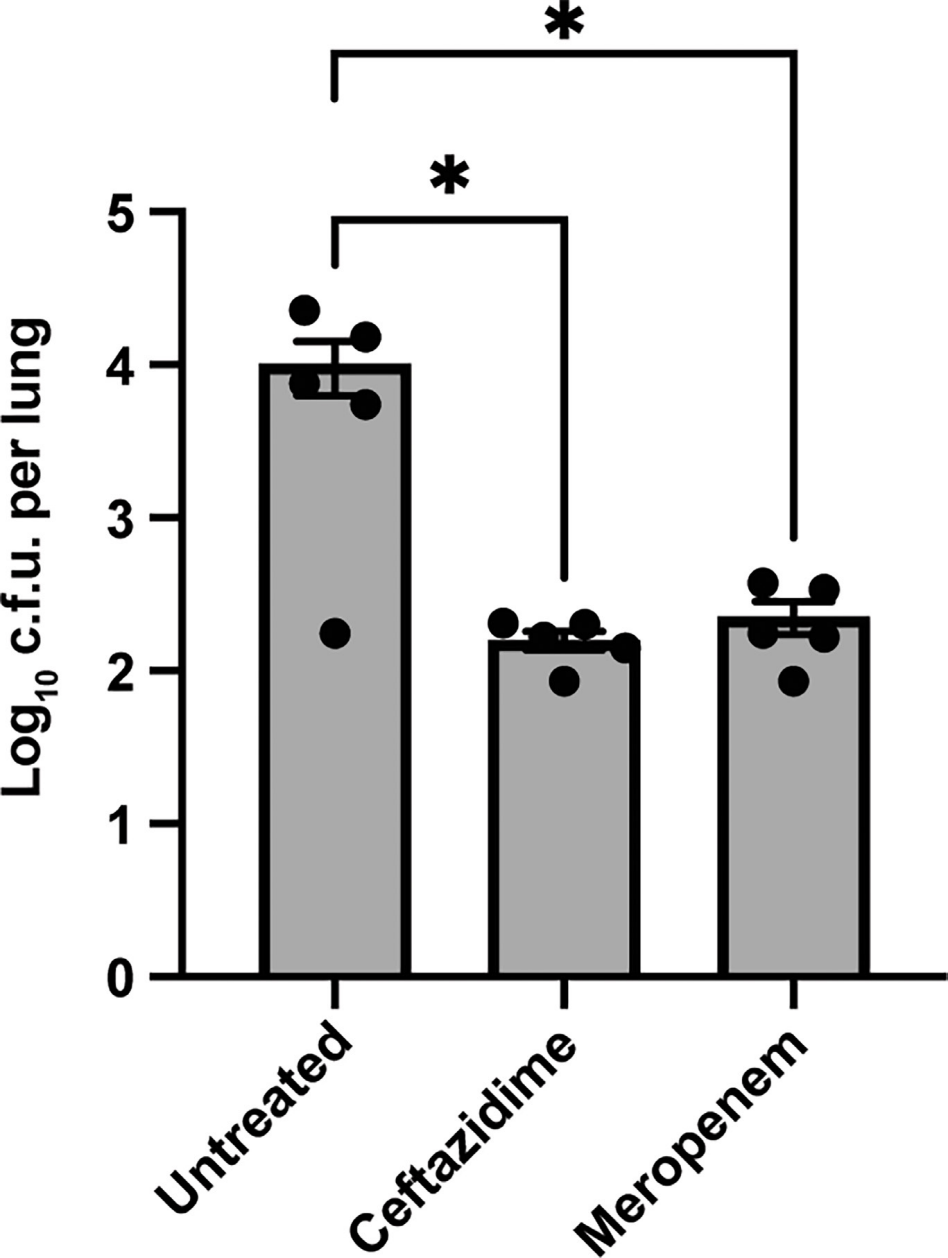
Ceftazidime and meropenem reduce but do not eradicate AU0728 infection in C3HeB/FeJ mice. Mice were treated with ceftazidime or meropenem (144 mg kg^−1^, intraperitoneally, twice at a 6 h interval) beginning 24 h p.i. Lungs were harvested 24 h after the first dose. Bars represent mean±sem; individual mice are shown as dots. *P* values were calculated using one-way ANOVA with Dunnett’s post-test versus untreated controls. *p < 0.05

At 48 h p.i., untreated mice exhibited a lung burden of 3.68±0.84 log₁₀ c.f.u. Treatment with ceftazidime or meropenem reduced lung bacterial burden to 2.18±0.16 and 2.30±0.26 log₁₀ c.f.u., respectively (~1.4–1.5-log reduction; ceftazidime vs. untreated: *P*=0.0163; meropenem vs. untreated: *P*=0.0170; Fig. 7). While both antibiotics significantly reduced bacterial burden, neither sterilized the lungs. These findings indicate that AU0728 persistence in C3HeB/FeJ mice cannot be attributed to intrinsic antibiotic resistance and support the utility of this model for evaluating therapeutic strategies aimed at reducing, but not trivially eliminating, pulmonary *B. cenocepacia* infection.

## Discussion

Developing chronic pulmonary infection models using human pathogens in immunocompetent mice remains a major challenge, largely due to the host’s rapid clearance of bacteria. Several strategies have been employed to model Bcc infection *in vivo*, including the use of gp91^phox^ knock-out mice that recapitulate CGD [21], administration of cyclophosphamide to induce leucopenia [22] and surgical instillation of agarose beads embedded with bacteria into the airways [23]. Each approach has notable limitations: the use of immunodeficient or genetically modified mice may limit generalizability; chemotherapeutic regimens introduce systemic toxicity; and surgical delivery is technically demanding and low-throughput. Moreover, these models typically support only short-term infection, limiting their utility for studying chronic infection or therapeutic interventions. A scalable, immunocompetent mouse model that supports sustained infection in at least some Bcc strains could provide critical insights into strain-specific virulence and therapeutic responses.

A promising candidate to address this modelling gap is the C3HeB/FeJ mouse strain, which carries a recessive allele at the sst1 locus (sst1^s^) and lacks expression of the macrophage-specific transcriptional regulator Ipr1 [13,14, 24]. This genetic profile impairs macrophage control of intracellular bacteria and renders C3HeB/FeJ mice susceptible to infections that are typically cleared in other immunocompetent strains. Following infection with *M. tuberculosis*, these mice develop necrotic lung lesions not observed in C57BL/6 or BALB/c mice. C3HeB/FeJ mice are also permissive to other intracellular pathogens, including *M. avium* [17], *L. monocytogenes* [15] and *C. pneumoniae* [16], suggesting that they may broadly support chronic infections where intracellular persistence is critical. However, to our knowledge, Bcc pathogenesis has not previously been studied in this model.

In this study, we used the C3HeB/FeJ mouse strain to establish a chronic pulmonary infection model for *B. cenocepacia*. A clinical isolate, AU0728, produced stable, non-sterilizing infection in the lungs, spleen and liver over a 42-day period without inducing necrotic lesions. However, a subset of infected animals exhibited signs of clinical deterioration and required early euthanasia, suggesting that host responses to persistent infection were heterogeneous. In contrast, other clinical isolates, including one additional *B. cenocepacia* strain and two *B. multivorans* strains, were rapidly cleared in this model. Clinical outcomes among CF patients infected with Bcc are notoriously heterogeneous; some individuals exhibit long-term, asymptomatic carriage, while others develop rapid necrotizing pneumonia and septicaemia, even when infected with the same strain [25]. These results suggest that chronic infection is not a general feature of Bcc species in this host but instead depends on strain-specific virulence traits in combination with host factors. The C3HeB/FeJ mouse thus provides a tractable system to investigate both host-intrinsic immune deficits and bacterial features that contribute to variable persistence and disease progression.

The hypersusceptibility of C3HeB/FeJ mice has been linked to defective IFN-γ–dependent macrophage activation. In *L. monocytogenes* infection, macrophages from sst1^R^ congenic C3HeB/FeJ mice exhibited stronger bactericidal activity than sst1^s^ controls, and this difference was abolished by IFN-γ blockade [15]. IFN-γ, a type II interferon, plays a central role in activating macrophages to produce pro-inflammatory cytokines, reactive oxygen and nitrogen species and other antimicrobial effectors [26]. Mice lacking IFN-γ are highly susceptible to a range of intracellular pathogens, including *Salmonella typhimurium* [27], *L. monocytogenes* [28] and *M. tuberculosis* [29]. In our study, IFN-γ–primed macrophages from C57BL/6 mice exhibited significant killing of *B. cenocepacia* AU0728, whereas C3HeB/FeJ macrophages failed to reduce intracellular burden. This defect does not appear to result from insufficient cytokine availability, as C3HeB/FeJ mice produce equal or higher levels of systemic IFN-γ compared to C57BL/6 mice in both viral [30] and mycobacterial [31] infections. Instead, our results suggest a cell-intrinsic defect in IFN-γ responsiveness or downstream signalling in C3HeB/FeJ macrophages.

This impaired responsiveness may be directly attributable to the absence of *Ipr1* expression. *Ipr1*, encoded within the sst1 locus, is an interferon-inducible nuclear protein that regulates multiple antibacterial pathways [13,14, 24]. IFN-γ stimulation upregulates *Ipr1* expression in macrophages, which in turn increases transcription of key cytokine receptors such as *Ifngr1* and *Tnfrsf1a* [32,33]. This feedback loop enhances macrophage responsiveness to IFN-γ and TNF-α. In sst1^R^ macrophages, *Ipr1* is expressed even under resting conditions and is further induced during infection. In contrast, sst1^s^ macrophages, including those from C3HeB/FeJ mice, fail to express full-length *Ipr1* transcripts under basal conditions or following IFN-γ stimulation [14]. The resulting disruption in IFN-γ signal transduction likely underlies the impaired antimicrobial response observed in AU0728-infected C3HeB/FeJ macrophages.

To further probe host factors associated with impaired bacterial control, we examined macrophage cytokine production and cell-death responses following *B. cenocepacia* AU0728 infection. Mixed-effects modelling revealed that IFN-γ pretreatment did not substantially alter the initiation of apoptotic signalling, which occurred rapidly and transiently in both strains. While C3HeB/FeJ macrophages exhibited a reduced magnitude of early apoptotic signalling compared with C57BL/6 macrophages, this defect was not rescued by IFN-γ pretreatment and did not fully account for differences in intracellular bacterial control. In contrast, late-stage membrane permeabilization increased progressively over time and exhibited strain- and IFN-γ–dependent modulation at later stages of infection. IFN-γ significantly influenced late fluorescence responses in infected macrophages, with strain-specific divergence emerging at 40 h p.i. Together, these data indicate that macrophage death during *B. cenocepacia* infection is governed by temporally ordered and differentially regulated phases, rather than a simple shift between apoptotic and necrotic fates.

The differential persistence of AU0728 may also reflect host antimicrobial mechanisms that were not directly assessed in this study. IFN-γ–activated macrophages produce ROS, which have been implicated in the killing of intracellular *Burkholderia* species [34], and inflammatory cell-death pathways such as pyroptosis may further contribute to intracellular clearance [35]. Beyond cell-intrinsic defences, complement-mediated bacterial killing could differentially influence the survival of distinct Bcc isolates *in vivo* [36]. Future studies will be needed to determine how these mechanisms interact with strain-specific bacterial traits and the sst1-dependent macrophage defect to shape susceptibility to *B. cenocepacia* infection in C3HeB/FeJ mice.

Only one of the four clinical Bcc isolates we tested, *B. cenocepacia* AU0728, was capable of sustaining infection in C3HeB/FeJ mice, while the others, including one additional *B. cenocepacia* and two *B. multivorans* strains, were rapidly cleared. This finding suggests that the ability to establish persistent intracellular infection is not a general feature of either species but instead depends on strain-level differences in virulence in combination with host factors. Importantly, the observation that persistence was limited to a single isolate highlights a key limitation of the current study and indicates that additional isolates will need to be evaluated to determine the broader applicability of C3HeB/FeJ mice as a model for chronic Bcc infection. Nevertheless, this behaviour aligns with multiple studies reporting high phenotypic variability among Bcc isolates and large variation in clinical manifestations of Bcc infections. In a longitudinal study of chronic CF infections, 215 isolates collected from 16 individuals showed extensive diversity in traits such as mucoidy, virulence and biofilm formation [37]. Even closely related strains, such as H111, K56-2 and J2315, differ significantly in their behaviour in model systems. For example, J2315 is less virulent than K56-2 in *Candida elegans* and *Galleria mellonella* [38,39], and although H111 and K56-2 showed comparable persistence in a rat agar bead model, only K56-2 induced extensive inflammation [39]. Similarly, isolate B17 (an ET12 strain) is more persistent than ATCC 25416 and provokes a stronger immune response in CFTR knockout mice [40].

Several molecular mechanisms may underlie these differences in Bcc strain behaviour and the increased susceptibility of C3HeB/FeJ to AU0728, though these were not directly examined in our study. Structural variation in the O-antigen of LPS can influence phagocytic uptake and immune recognition of both *B. cenocepacia* and *B. multivorans* isolates [41,42], and reduced glycosylation of surface molecules may aid in immune evasion and long-term persistence [43]. In addition, virulence factors delivered via type III, IV and VI secretion systems contribute to intracellular survival and host-cell manipulation in Bcc [44,47]. Disruption of these systems alters virulence in multiple infection models. Finally, antibiotic resistance can itself modulate virulence gene expression [48,51]. AU0728 exhibits higher resistance to fluoroquinolones and lower resistance to *β*-lactams than other isolates tested.

Our model demonstrates antibiotic responsiveness: C3HeB/FeJ mice infected with AU0728 showed a ~1.5-log reduction in lung bacterial burden following treatment with either ceftazidime or meropenem, although neither regimen achieved sterilization. While this single *in vivo* treatment experiment does not establish the broader utility of the model for evaluating therapeutics against *B. cenocepacia*, it provides initial proof of concept that bacterial burden in this system is pharmacologically modifiable in the context of persistent infection. The partial efficacy of ceftazidime is noteworthy, as this *β*-lactam exhibits limited intracellular penetration and is typically more effective against extracellular bacteria [34]. In contrast, meropenem possesses both extracellular and intracellular activity, which may explain its similar *in vivo* performance [52,53]. That ceftazidime nonetheless reduced lung burden suggests that an extracellular component contributes to AU0728 persistence in this model. Alternatively, ceftazidime may act synergistically with IFN-γ signalling. In *Burkholderia pseudomallei* infection, combination treatment with ceftazidime and IFN-γ has been shown to enhance bacterial clearance [54]. A similar synergistic effect could underlie the reduction in AU0728 burden observed in our study. *In vitro* assays often fail to predict clinical outcomes in Bcc infection, due to the absence of host immune components that influence bacterial clearance [55]. Our *in vivo* experiment begins to address this gap by integrating both host immune context and strain-level variation. Future studies incorporating extended treatment regimens will be needed to determine whether sustained antibiotic therapy can resolve the inflammatory pathology observed in chronically infected C3HeB/FeJ lungs.

In conclusion, this study describes a pulmonary infection model for *B. cenocepacia* in immunocompetent C3HeB/FeJ mice. Among the four clinical Bcc isolates tested (two *B. cenocepacia* and two *B. multivorans* strains), only *B. cenocepacia* AU0728 sustained long-term infection, highlighting the strain-specific nature of Bcc pathogenesis in this host. These findings emphasize the importance of both bacterial virulence factors and host immune responses, including macrophage-intrinsic signalling pathways, in determining infection outcomes. While this model provides initial proof of concept for studying chronic *B. cenocepacia* infection, further evaluation with additional clinical isolates and extended therapeutic studies will be necessary to establish its broader utility for testing antimicrobial interventions. Ongoing efforts to genetically characterize AU0728 and comparator strains will help elucidate the bacterial determinants that contribute to persistence and immune evasion *in vivo*.

## Supplementary material

10.1099/jmm.0.002153 Uncited Supplementary Material 1.

## Abbreviations

Bcc: *Burkholderia cepacia* complex
BMDMs: bone marrow–derived macrophages
CF: cystic fibrosis
CGD: chronic granulomatous disease
DMEM: Dulbecco’s modified eagle medium
M-CSF: macrophage colony-stimulating factor
MOI: multiplicity of infection
OD₆₀₀: OD at 600 nm
ROS: reactive oxygen species
