# Supplementary material for: Strain-specific persistence of Burkholderia cenocepacia in the C3HeB/FeJ mouse model of pulmonary infection
Source: J Med Microbiol. 2026 Apr 9;75(4):002153. doi: 10.1099/jmm.0.002153 (PMC13065297; doi:10.1099/jmm.0.002153)
Supplement: Uncited Supplementary Material 1. [file jmm-75-02153-s001.pdf]

Supplementary Information

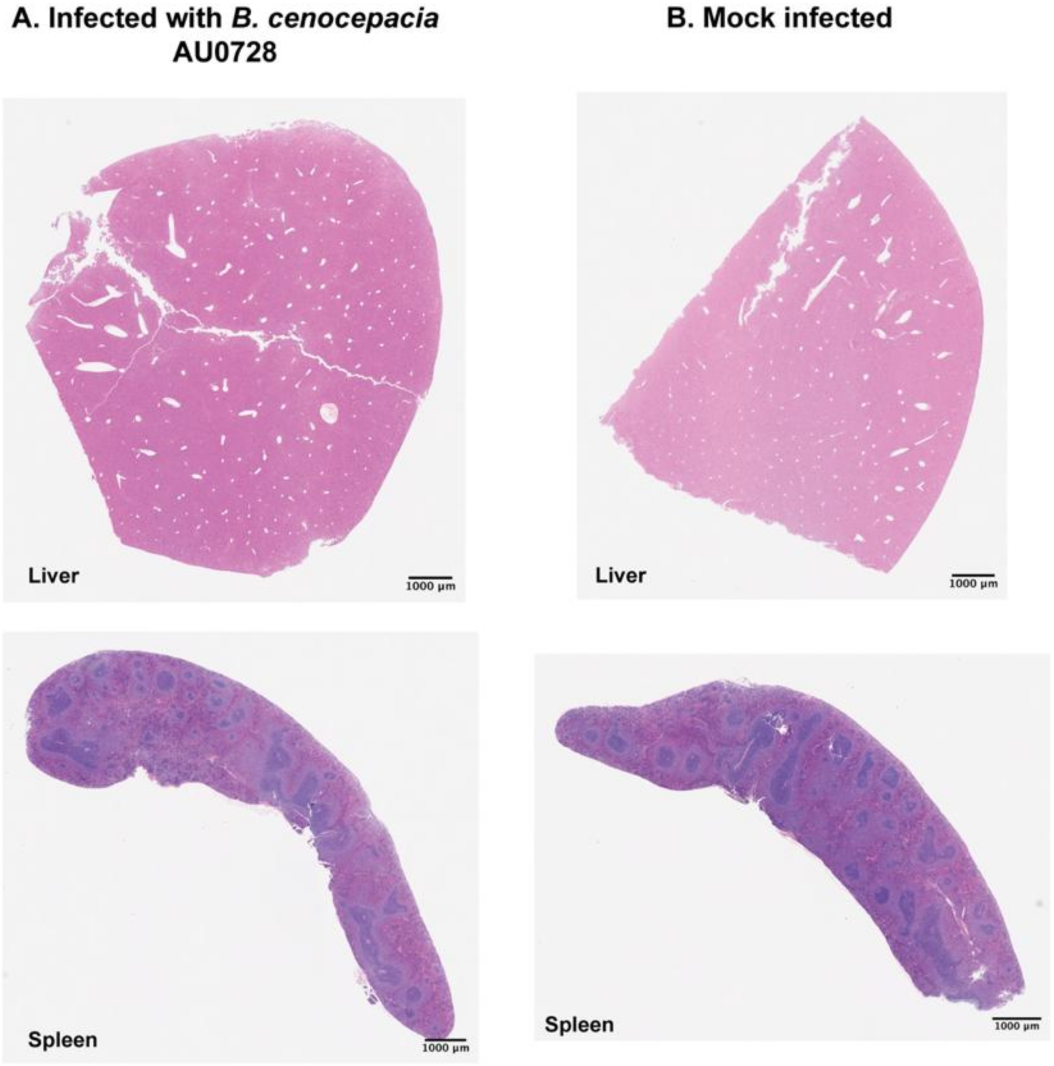

Figure S1. Histological comparison of liver and spleen tissue at day 42 post-infection. Hematoxylin and eosin (H&E)-stained sections from mice infected with *B. cenocepacia* AU0728 (A) and mock-infected controls (B) reveal no overt histopathologic differences in the liver (upper panel) or spleen (lower panel). Scale bars: 1000 µm

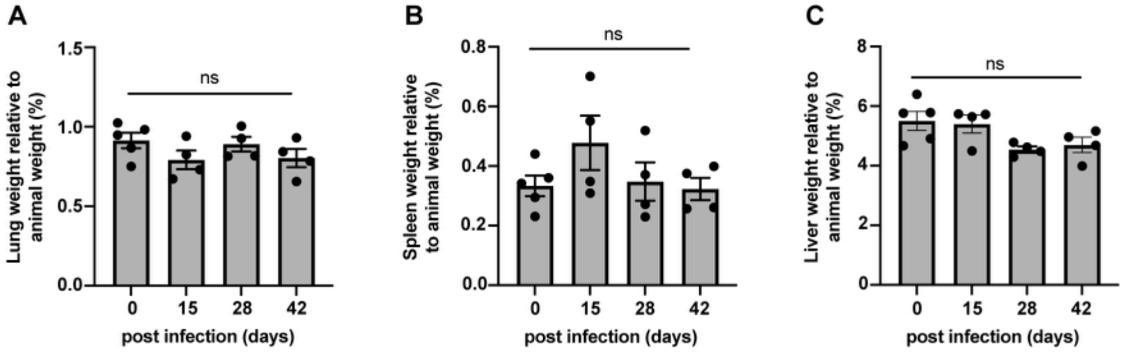

Figure S2. Organ weights remain stable during chronic AU0728 infection. (A–C) Relative weights of lung, spleen, and liver (normalized to total body weight) at baseline, day 15, day 28, and day 42 post-infection (p.i.) in C3HeB/FeJ mice. No significant differences were observed compared to baseline (one-way ANOVA with Dunnett's multiple comparisons test). Bars indicate mean ± SEM; dots represent individual mice.

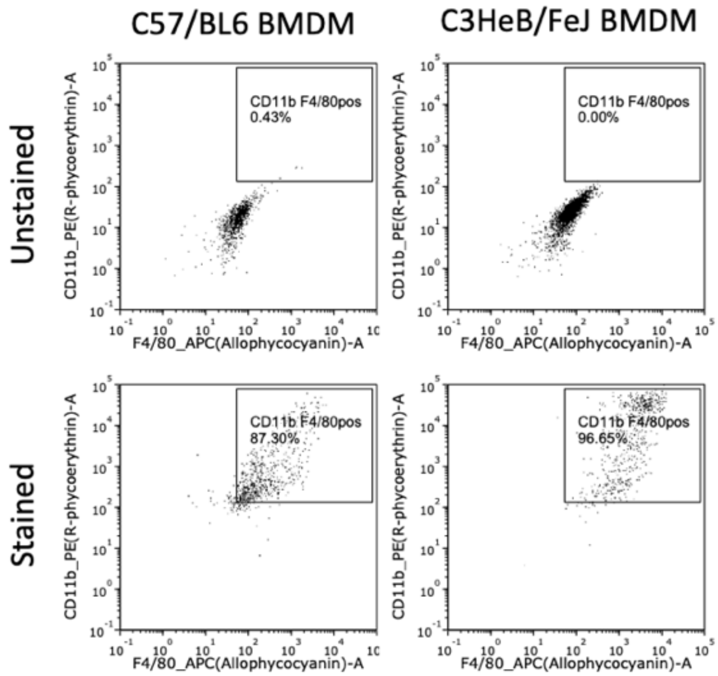

Figure S3. Flow cytometry confirms successful BMDM differentiation. CD11b and F4/80 double-positive macrophages were detected by flow cytometry in macrophage cultures differentiated from bone-marrow derived monocytes from C57BL/6 and C3HeB/FeJ mice. Unstained controls are shown for each strain
